# Molecular detection of *Angiostrongylus vasorum* in gastropods in Surrey, UK

**DOI:** 10.1007/s00436-018-6191-1

**Published:** 2019-01-26

**Authors:** L. Hicklenton, M. Betson

**Affiliations:** School of Veterinary Medicine, University of Surrey, Guildford, GU2 7AL UK

**Keywords:** *Angiostrongylus vasorum*, Gastropod, *Deroceras invadens*, *Arion rufus*

## Abstract

Surrey, a county in southern England, is a hot spot for angiostrongylosis in domestic dogs but there have been no investigations into the intermediate hosts of *Angiostrongylus vasorum* in this area. This study aimed to determine the prevalence of *A. vasorum* in gastropods in Guildford, the most populous town in Surrey, and to ascertain which gastropod species can act as intermediate hosts for *A. vasorum*. Gastropods (*n* = 97) were sampled in six locations, representing urban, suburban and rural environments, and identified to species based on morphological features. A PCR assay was used to detect *A. vasorum* DNA in gastropod tissue and the species of infected specimens was confirmed by sequencing of mitochondrial genes. 4.1% (4/97) of sampled gastropods and 9.1% (4/44) of sampled slugs were *A. vasorum* positive. Infected gastropod species were *Arion rufus* (*n* = 3) and *Deroceras invadens* (*n* = 1), the first description of the latter species as a potential intermediate host for *A. vasorum*. Two infected slugs were sampled in urban environments and two in suburban environments. The results demonstrate that there is a risk of transmission of *A. vasorum* to domestic dogs from the gastropod population in urban and suburban areas of Guildford.

**Electronic supplementary material:**

The online version of this article (10.1007/s00436-018-6191-1) contains supplementary material, which is available to authorized users.

## Introduction

The lungworm *Angiostrongylus vasorum* causes the serious and potentially fatal disease angiostrongylosis in domestic dogs (Elsheikha et al. 2014). It can also infect wild carnivores, such as red foxes, which are important reservoir hosts. In the UK, there are established hyperendemic foci of the parasite in Wales and southeast England and newer endemic foci in the north of England and Scotland (Helm et al. 2009; Kirk et al. 2014). *Angiostrongylus vasorum* has an indirect lifecycle with larval development taking place within a gastropod intermediate host. Dogs are infected through ingestion of gastropods carrying infective L3 larvae. A number of gastropod species have been implicated as intermediate hosts for *A. vasorum* in the UK (Aziz et al. 2016; Helm et al. 2015; Jefferies et al. 2009; Patel et al. 2014). Surrey, a county in southern England (population 1.1 million), is a hot spot for angiostrongylosis in domestic dogs (Chapman et al. 2004; Kirk et al. 2014). To date, there have been no investigations into the intermediate hosts of *A. vasorum* in this area. This study aimed to determine the prevalence of *A. vasorum* in gastropods in the area of Guildford, the most populous town in Surrey, and to ascertain which gastropod species can act as intermediate hosts for *A. vasorum*.

## Materials and methods

A total of 97 gastropods were collected from six sites around Guildford in Autumn 2016. Collection sites were chosen based on accessibility and to represent urban, sub-urban and rural environments. Urban sites were in built up areas such as housing estates along the sides of paths and roads. Suburban sites were in open areas such as parks surrounded by built up areas. Rural sites were away from built up areas and included woodland. Gastropods were collected using forceps (and a torch if needed) between the hours of 17:00 and 11:30 and were found by looking on the ground, in hedgerows and in the undergrowth. Gastropods were identified to species using morphological keys (Kerney and Cameron 1994; Rowson et al. 2014a) and then frozen at − 20 °C.

DNA was extracted from 10 to 20 mg of gastropod foot tissue using the DNeasy Blood and Tissue kit (Qiagen). *Angiostrongylus vasorum* DNA was detected by PCR using the AvasF and AvasR primers (Helm et al. 2009). For molecular identification of *A. vasorum-*positive gastropods, cytochrome c oxidase subunit I (COI) and 16S rDNA genes were amplified using LCO1490 and HCO2198 (Folmer et al. 1994) and 16S-1 and 16S-2 (Barr et al. 2009) primers, respectively. Each 25 μl PCR reaction contained 12.5 μl MyTaq HS Red mix (Bioline, London, UK), 0.4 pmole of the forward and reverse primers and 2 μl of eluted DNA. Cycling conditions were as follows: 95 °C for 1 min, then 30 cycles of 95 °C for 15 s, 55 °C (Avas)/42 °C (CO1)/50 °C (16S) for 15 s and 72 °C for 15 s. PCR products were submitted for Sanger sequencing. BLAST searches of GenBank indicated that *A. vasorum* positive gastropods belonged to the larger Arionidae (*n* = 3) and Agriolimacidae (*n* = 1) families. 16S sequences representing all clades of the larger Arionidae were aligned with 16S sequences of the Arionidae specimens using CLUSTALW in MEGA v6 (Tamura et al. 2007). The alignment was used to construct a neighbour joining tree based on Kimura 2-parameter distance (Rowson et al. 2014a). Bootstrapping (1000 replicates) was carried out to test branch reliability. Similar analysis was carried out for the Agriolimacidae specimen using COI sequences.

## Results and discussion

The 97 gastropods collected were classified into nine families, 15 genera and 21 species (see Supplementary Table). Differentiation between certain species (e.g., *Cepaea hortensis and C. nemoralis*) was not always possible based on morphology. There was a significant association between gastropod family and location type (Fisher’s exact test *p* < 0.0001), likely due to local ecological factors such as vegetation which favour particular families. Similar slug species were sampled in Swansea and Bristol (Aziz et al. 2016), although the relative proportions of species vary in the different locations. Interestingly, some *A. vasorum*-negative gastropod species sampled in Guildford have been implicated as intermediate hosts for *A. vasorum*, suggesting that they could also play a role in local transmission (Ferdushy and Hasan 2010; Helm et al. 2015).

Four (4.1%) of the 97 gastropods examined were positive for *A. vasorum* infection by PCR. All were slugs, meaning that 9.1% (4/44) of slugs were *A. vasorum* positive. The *A. vasorum* prevalence in gastropods is comparable to that recorded in Glasgow (Helm et al. 2015). The prevalence in slugs is higher than in London (Patel et al. 2014) and in Bristol, and lower than in Swansea (Aziz et al. 2016). Explanations for geographical differences could include variations in gastropod density and species composition, effects of local environment on parasite transmission and methods used for *A. vasorum* detection.

**Fig. 1:**
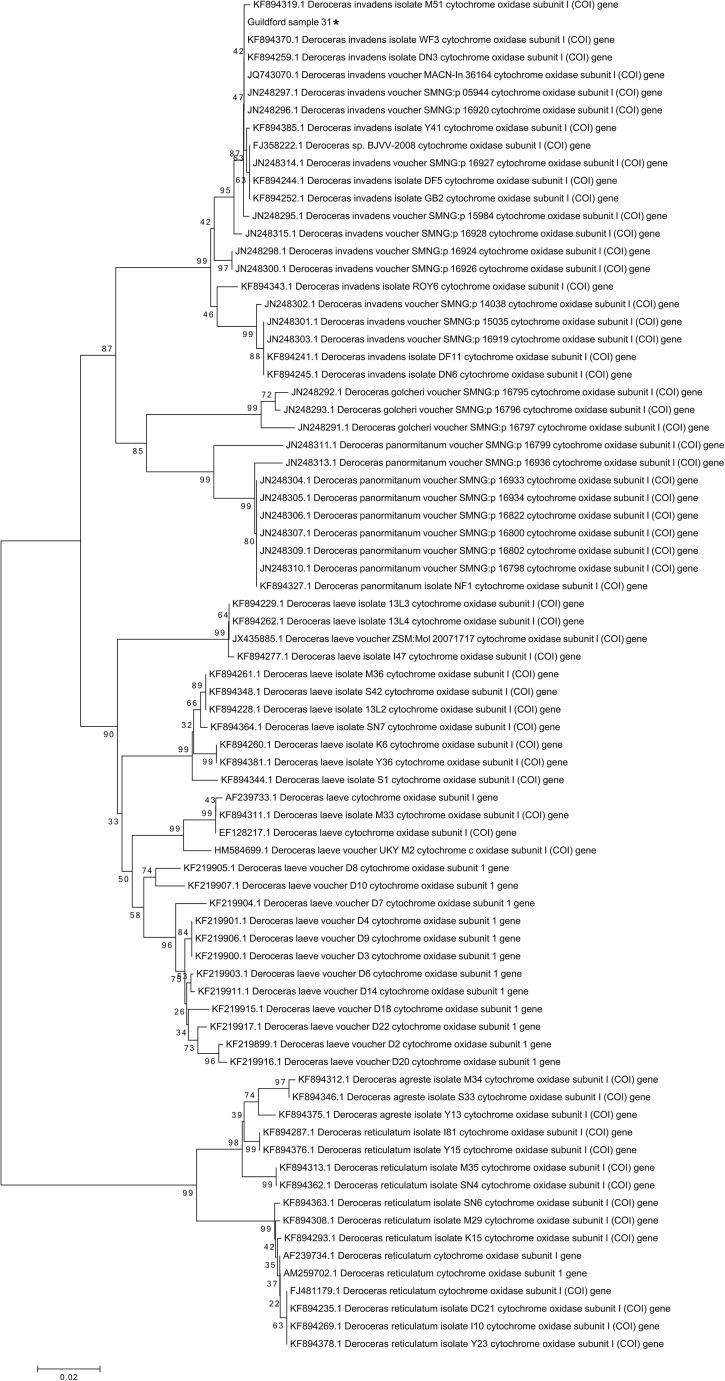
Neighbour joining tree based on cytochrome oxidase I (COI) sequences representing all clades of the Agriolimacidae family. Values next to branches indicate percentage bootstrap support. The asterisk (*) indicates the Agriolimacidae specimen collected in this study

Sequencing of mitochondrial gene regions of *A. vasorum*-positive gastropods revealed that three belonged to the larger Arionidae family and the one to the Agriolimacidae family. Phylogenetic analysis of 16S sequences revealed that the three Arionidae gastropods belonged to the European *Arion rufus* clade (Supplementary Fig. 1), whereas similar analysis of COI sequences demonstrated that the *A. vasorum*-positive Agriolimacidae specimen belonged to the *Dercoceras invadens* clade (Fig. 1) (Rowson 2016). Two positive *Ar. rufus* specimens were found in urban environments. The two other positive specimens were found in suburban environments. Infected *Ar. rufus* slugs have been reported in the UK and Europe (Aziz et al. 2016; Eckert and Lammler 1972; Ferdushy et al. 2009; Guilhon and Cens 1973; Helm et al. 2015; Patel et al. 2014). *Arion rufus* is a large slug species and may be more likely to be ingested by dogs than smaller species. This study represents the first report of *D. invadens* as a potential intermediate host for *A. vasorum*, although natural infections of other *Deroceras* species have been described (Ferdushy and Hasan 2010; Jefferies et al. 2009; Lange et al. 2018). *Deroceras invadens* is widely distributed throughout the UK and Europe, typically occurring in disturbed, urban and roadside sites (Rowson et al. 2014b), suggesting ample opportunities for dogs to encounter this species.

In conclusion, there is a risk of transmission of *A. vasorum* to domestic dogs from the gastropod population in urban and suburban areas of Guildford. In addition, a new potential intermediate host for *A. vasorum* has been identified. Further work is needed to confirm whether there are differences in prevalence between urban, suburban and rural areas and investigate seasonal variations in transmission risk.

## Electronic supplementary materials

Supplementary Fig. 1 Neighbour joining tree based on 16S sequences representing all clades of the larger Arionidae. Values next to branches indicate percentage bootstrap support. Specimens collected in this study are labelled “Guildford sample”. (PDF 19 kb)

Supplementary Table 1 (PDF 279 kb)
