# Supplementary figures and images for: Molecular detection of Angiostrongylus vasorum in gastropods in Surrey, UK
Source: Parasitol Res. 2019 Jan 26;118(3):1051–4. doi: 10.1007/s00436-018-6191-1 (PMC6514071; doi:10.1007/s00436-018-6191-1)

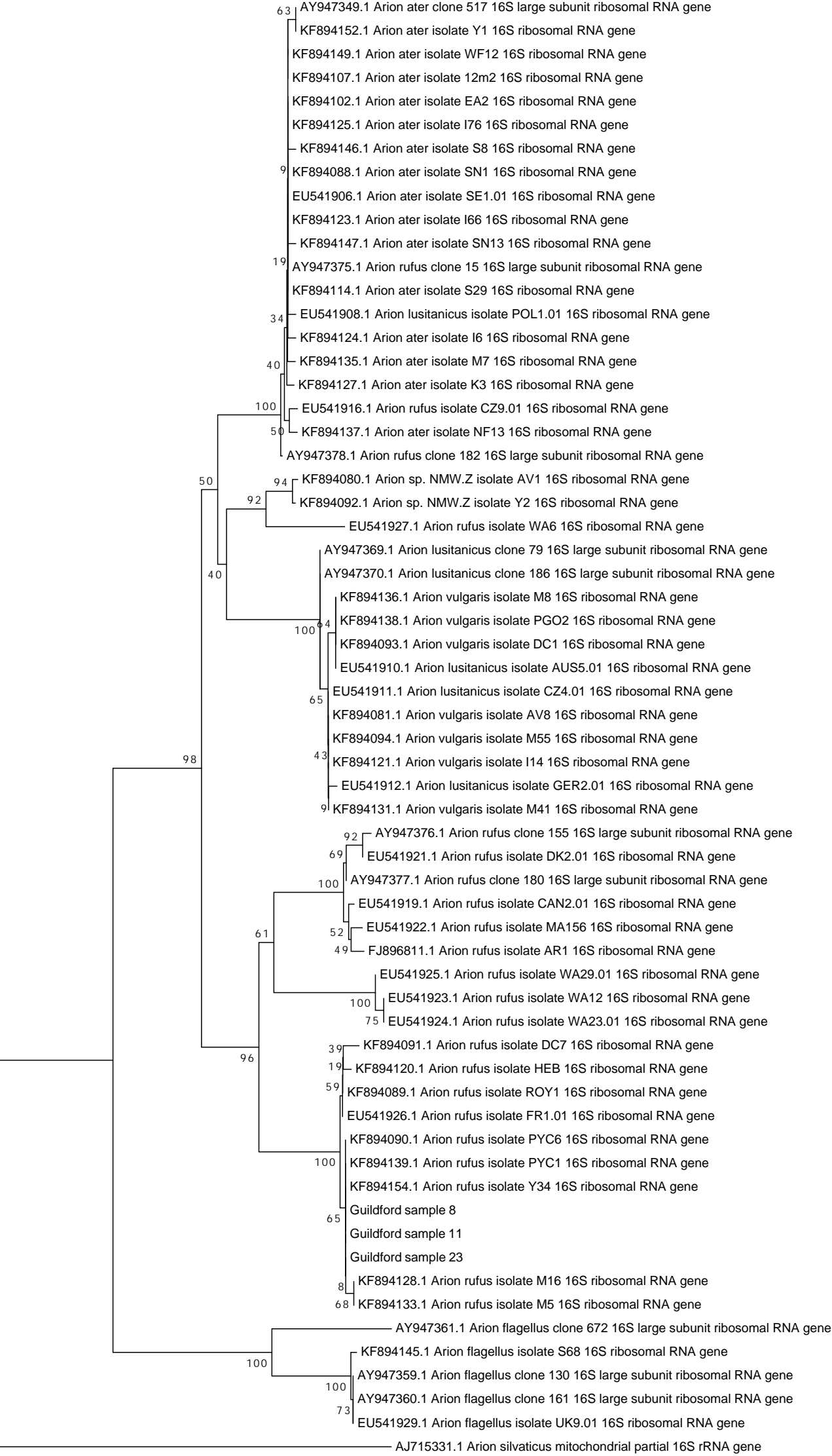

0.02

Supplement: Supplementary file 1 — Neighbour joining tree based on 16S sequences representing all clades of the larger Arionidae. Values next to branches indicate percentage bootstrap support. Specimens collected in this study are labelled “Guildford sample”. (PDF 19 kb) [file 436_2018_6191_MOESM1_ESM.pdf]
