# Supplementary material for: Molecular detection of Angiostrongylus vasorum in gastropods in Surrey, UK
Source: Parasitol Res. 2019 Jan 26;118(3):1051–4. doi: 10.1007/s00436-018-6191-1 (PMC6514071; doi:10.1007/s00436-018-6191-1)
Supplement: Supplementary file 2 — (PDF 279 kb) [file 436_2018_6191_MOESM2_ESM.pdf]

Supplementary Table. Number of specimens of each gastropod species collected in urban, suburban and rural locations around Guildford, Surrey, UK in Autumn 2016

Type	Family	Species	Rural	Suburban	Urban	Total
Slug	Agriolimacidae	*Deroceras laeve*	0	0	2	2
		Deroceras reticulatum	1	2	0	3
	Arionidae	*Arion flagellus*	0	0	1	1
		Arion rufus	0	7	2	9
		Arion rufus/vulgaris/davies	0	2	1	3
		Arion (Kobeltia) hortensis	1	0	5	6
		Arion (Kobeltia) intermedius	0	0	1	1
		Arion (Kobeltia) owenii	0	0	1	1
		Arion spp.	0	0	1	1
	Limacidae	*Lehmannia marginata*	1	0	0	1
		Limacus maculatus	0	0	3	3
	Milacidae	*Tandonia budapestensis*	0	0	2	2
	Unknown	-	1	6	4	11
Snail	Euconulidae	*Euconulus fulvus*	1	0	0	1
	Helicidae	*Arianta arbustorum*	2	1	0	3
		Cornu aspersum	0	0	4	4
		Cepaea hortensis	3	11	4	18
		Cepaea hortensis/nemoralis	0	5	3	8
		Cepaea hortensis/ Arianta arbustorum	0	2	0	2
	Hygromidae	*Monacha cantiana*	1	0	0	1
		Hygromia cinctella	0	1	0	1
		Trichia hispida	4	0	0	4
		Trichia striolata	0	0	1	1
		Perforatella subrufescens	0	2	0	2
	Oxychilidae	*Oxychilus* spp.	5	2	0	7
	Succineidae	*Succinea putris/pfeifferi*	1	0	0	1
**Total**			**21**	**41**	**35**	**97**
